# Supplementary figures and images for: PhyteByte: identification of foods containing compounds with specific pharmacological properties
Source: BMC Bioinformatics. 2020 Jun 10;21:238. doi: 10.1186/s12859-020-03582-7 (PMC7288679; doi:10.1186/s12859-020-03582-7)

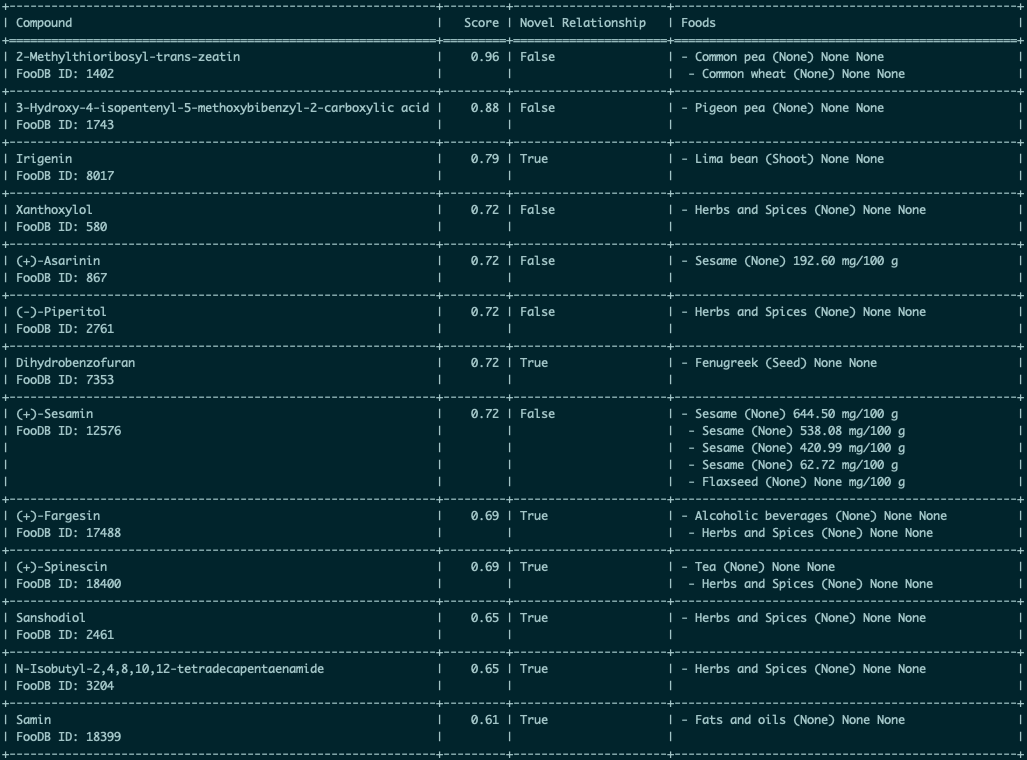

Supplement: Supplementary file 2 — Additional file 2. [file 12859_2020_3582_MOESM2_ESM.png]
